# Integrated network modeling approach defines key metabolic responses of soil microbiomes to perturbations

**DOI:** 10.1038/s41598-020-67878-7

**Published:** 2020-07-02

**Authors:** Ryan S. McClure, Joon-Yong Lee, Taniya Roy Chowdhury, Eric M. Bottos, Richard Allen White, Young-Mo Kim, Carrie D. Nicora, Thomas O. Metz, Kirsten S. Hofmockel, Janet K. Jansson, Hyun-Seob Song

**Affiliations:** 1 Biological Sciences Division, Pacific Northwest National Laboratory, Richland, WA USA; 2 Department of Ecology, Evolution and Organismal Biology, Iowa State University, Ames, IA USA; 3 Department of Biological Systems Engineering, University of Nebraska-Lincoln, Lincoln, NE USA; 4 Nebraska Food for Health Center, Department of Food Science and Technology, University of Nebraska-Lincoln, Lincoln, NE USA; 5 Present Address: Department of Environmental Science and Technology, University of Maryland, College Park, MD USA; 6 Present Address: Department of Biological Sciences, Thompson Rivers University, Kamloops, BC Canada; 7 Present Address: Department of Plant Pathology, Washington State University, Pullman, WA USA

**Keywords:** Computational biology and bioinformatics, Environmental sciences

## Abstract

The soil environment is constantly changing due to shifts in soil moisture, nutrient availability and other conditions. To contend with these changes, soil microorganisms have evolved a variety of ways to adapt to environmental perturbations, including regulation of gene expression. However, it is challenging to untangle the complex phenotypic response of the soil to environmental change, partly due to the absence of predictive modeling frameworks that can mechanistically link molecular-level changes in soil microorganisms to a community’s functional phenotypes (or metaphenome). Towards filling this gap, we performed a combined analysis of metabolic and gene co-expression networks to explore how the soil microbiome responded to changes in soil moisture and nutrient conditions and to determine which genes were expressed under a given condition. Our integrated modeling approach revealed previously unknown, but critically important aspects of the soil microbiomes’ response to environmental perturbations. Incorporation of metabolomic and transcriptomic data into metabolic reaction networks identified condition-specific signature genes that are uniquely associated with dry, wet, and glycine-amended conditions. A subsequent gene co-expression network analysis revealed that drought-associated genes occupied more central positions in a network model of the soil community, compared to the genes associated with wet, and glycine-amended conditions. These results indicate the occurrence of system-wide metabolic coordination when soil microbiomes cope with moisture or nutrient perturbations. Importantly, the approach that we demonstrate here to analyze large-scale multi-omics data from a natural soil environment is applicable to other microbiome systems for which multi-omics data are available.

## Introduction

Metabolic network reconstruction has been used as a common tool to model a broad range of biological systems, including single microorganisms^1,2^ as well as simplified communities of microbial species^3,4^. Concurrent with these applications, there have been several computational advances in integrating omics data into metabolic networks^5,6^. However, their extension to natural ecosystems such as soil microbiomes is currently challenging due to several factors, including the complexity of multi-omics data integration^7^, low data quality^8^, and the difficulty in constructing reliable metabolic network models. Our recent development of a new metabolic network-omics integration method (termed the Metabolite-Expression-Metabolic Network Integration for Pathway Identification and Selection; MEMPIS) was motivated to overcome these barriers^9^. The MEMPIS approach enabled identifying condition-specific metabolic reactions and genes by incorporating both metabolite and gene expression profiles. Key questions that subsequently arose but remain unanswered are how condition-specific genes are structurally connected to other genes and how central they are to the response of the soil microbiome as a whole. To address these questions, we aimed to integrate our previous work with a complementary gene interaction network model^10–12^.

Previous studies of soil gene expression profiles have examined how the soil responds to one or more conditions in isolation^13,14^. While these approaches can be useful for determining how the soil microbiome responds to specific conditions of interest, a high-level view of the system can only be obtained when all of the data is combined and instances of co-expression between genes across conditions can be viewed as a *network*. Networks of this type, where genes are linked based on co-expression, have been inferred for a number of prokaryotic and eukaryotic species^15,16^ but are just starting to be examined for communities consisting of multiple species^17^. Some studies have linked species in networks based on their co-abundance^18,19^. However, a network of genes based on co-expression can provide more detailed information about how specific pathways are related and which processes are central not only to specific conditions but to the biological system as a whole. Such approaches have previously been used to identify gene-to-gene connections (pointing to their centrality in the network and their importance to the system)^10,11^ and to show coordinated responses across conditions^17^.

Here, we used a general modeling platform that integrates metabolic and gene co-expression networks to reveal the fundamental relationships between condition-specific gene functions and their centralities in the soil microbiome. For this purpose, we created metabolic models using multi-omics data collected from a native prairie soil microbiome that was subjected to different perturbations, including changes in soil moisture and nutrient addition. Previously, we used MEMPIS to identify condition-specific genes and reactions in response to changes in soil moisture^9^. We showed that our metabolic network-based prediction of condition-specific genes is more sensitive and powerful compared to typical feature selection, for example those that only focus on genes that are up or downregulated when comparing pairs of conditions^20^. Here, we compared different environmental perturbations, including addition of nutrients to soil (glycine, a common root exudate^21,22^), with existing moisture perturbation data^9^ to infer gene co-expression networks. We aimed to determine the centrality of those genes identified by MEMPIS that responded to specific conditions (e.g., the degree to which the responding genes are linked to other genes and how critical they are to the structure of the network). This allowed us to address new hypotheses related to the importance of processes responding to certain conditions (wet, dry, and glycine addition) within a global network of the soil microbiome. This combination of network analyses presented here revealed that most genes associated with dry conditions occupied highly central positions in the network, more so than genes responding specifically to wet conditions or glycine amendment. Our integrative network approach offers a powerful way to interrogate the metaphenotypic response^23^ of complex and diverse microbial communities to a number of specific perturbations.

## Results

### Identification of signature genes and their functional implications in metabolic pathways

**Figure 1:**
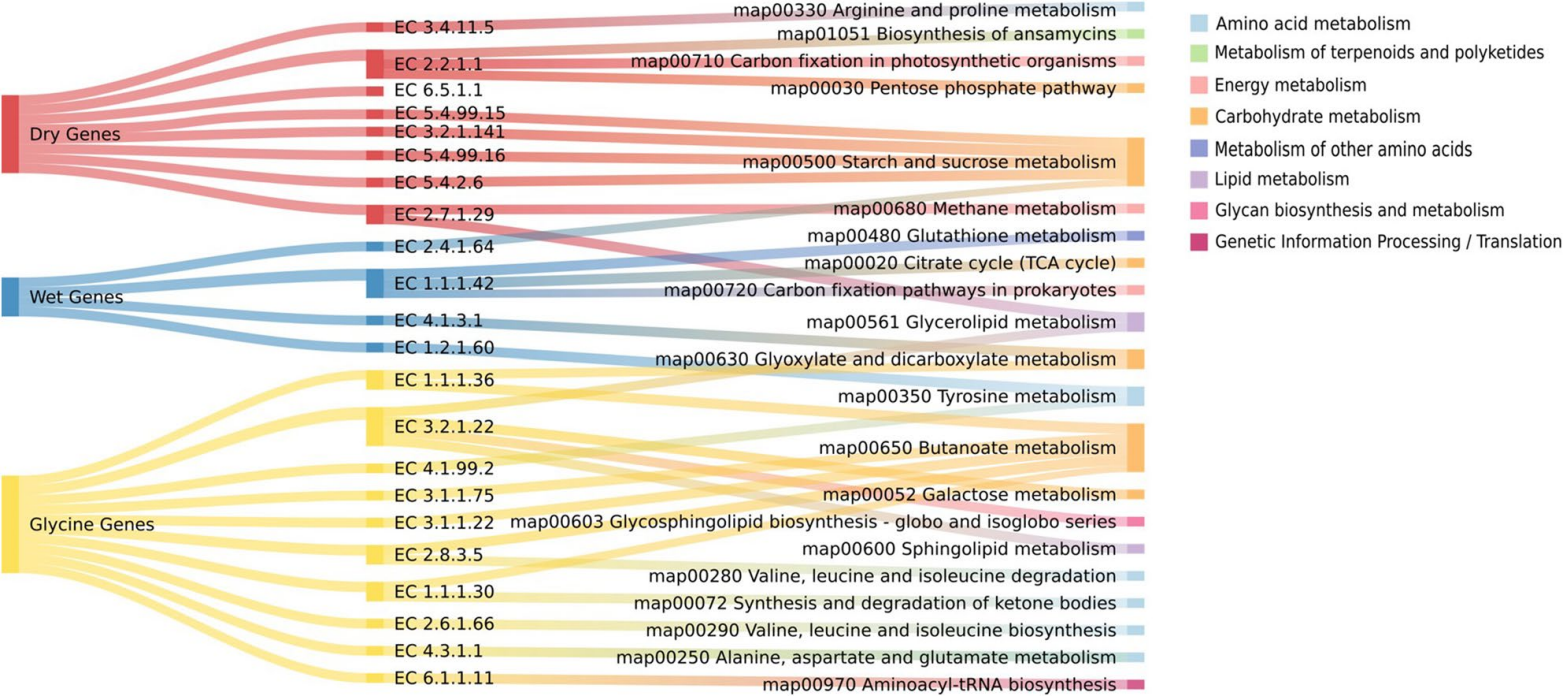
Condition-specific genes predicted from MEMPIS and the associated metabolic pathways. 21 condition-specific genes (except for EC 6.5.1.1) are broadly associated with 21 KEGG pathways with only a few overlaps (See Supp Table 1). The starch and sucrose metabolism pathways include four dry-associated genes and one wet gene, and the butanoate metabolism pathway includes five glycine genes. Overall, carbohydrate metabolism responds to both moisture and carbon amendments while glycine genes are associated with amino acid metabolism more so than other condition-specific genes.

Application of MEMPIS, an algorithm that simultaneously integrates metabolite and gene expression profiles into metabolic networks, led to the identification of microbial reactions and genes (referring to gene functions described by EC numbers derived from transcript sequences) that are *uniquely* associated with specific soil perturbations: dry, wet, and glycine-amended soils (Supp Table 1). Unique genes for each condition were defined as those predicted to be associated with only one specific perturbation condition. The number of uniquely responsive genes varied across the conditions, with 8, 4, and 10 unique genes for dry, wet, and glycine-amended conditions, respectively (Supp Table 1 and Fig. 1). In contrast with our previous study^9^ that focused only on moisture perturbations, the list of genes here was determined by including the results from glycine amendment. We note that, despite this additional perturbation dataset, the resulting unique genes for dry and wet conditions remained the same, indicating that the responses of the soil microbiome to water stress and nutrient perturbations were metabolically distinct.

**Table 1:** Centrality of genes in a transcriptomic network describing glycine and moisture amended soil.

E.C	Function	Betweenness	Degree
*Genes of high betweenness*			
2.4.1.18	1,4-alpha-glucan branching enzyme	0.0598	10
1.1.99.23	Cellobiose dehydrogenase	0.0588	8
6.2.1.17	Propionate–CoA ligase	0.0583	10
2.2.1.1	Glycoaldehyde transferase	0.0532	21
2.5.1.47	Cysteine synthase	0.0523	10
2.4.1.19	Cyclomaltodextrin glucanotransferase	0.0521	4
2.4.1.129	Peptidoglycan glycosyltransferase, Penicillin binding protein	0.0517	12
2.7.1.36	Mevalonate kinase	0.0496	10
2.4.1.119	Antibiotic biosynthesis	0.0455	9
3.1.1.58	N-acetylgalactosaminoglycan deacetylase	0.0454	7
*Genes of high degree*			
1.6.99.3	NADH dehydrogenase	0.0383	24
1.1.99.8	Pyranose dehydrogenase	0.0191	24
2.2.1.1	Glycoaldehyde transferase	0.0532	21
5.3.1.9	Glucose-6-phosphate isomerase	0.0118	21
1.9.3.1	Cytochrome-c oxidase	0.0353	20
1.10.2.2	Quinol–cytochrome-c reductase	0.0161	20
2.2.1.2	Dihydroxyacetone transferase	0.0331	19
5.99.1.2	DNA topoisomerase	0.0122	19
1.1.1.44	6-phosphogluconic carboxylase	0.0120	19
2.4.1.8	Maltose phosphorylase	0.0315	18

To understand the functional implications of condition-dependent unique genes that were expressed and identified in the data in Supp Table 1, we mapped predicted gene sets onto the KEGG reaction network. Many of the ‘dry-associated genes’ were found in the pathway for trehalose metabolism, part of sucrose and starch metabolism (Supp Fig. 1A and Supp Fig. 2A). By contrast, ‘wetting-associated genes’ were found sporadically across different reaction modules and located in isolation, making it difficult to identify connected reaction pathways as biochemical signatures. This prediction supports our previous work^9^ by reconfirming the activation of a set of dry-associated genes/reactions in the trehalose synthesis pathway even after newly incorporating glycine-amended data. Most of the unique glycine genes were involved in butanoate metabolism and connected reactions (Supp Fig. 1B and Supp Fig. 3). These genes included those encoding hydroxybutyrate dehydrogenase and poly(3-hydroxybutyrate) depolymerase that are related to the energy-storage and availability of nitrogen, phosphorus or oxygen in the environment^24–26^. We also found that genes primarily associated with fatty acid synthesis were commonly predicted under all three conditions (Supp Fig. 4).

**Figure 2:**
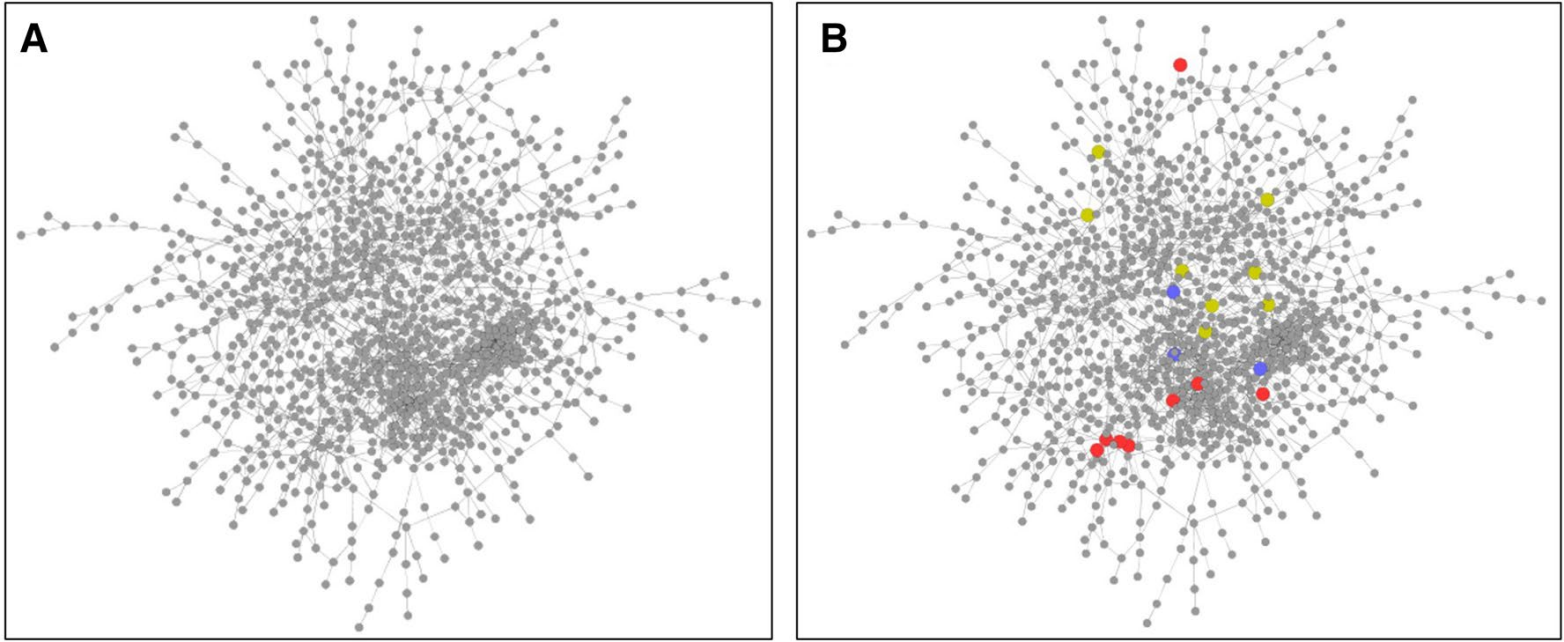
Gene co-expression network analysis of Kansas soil. (**A**) A gene co-expression network was inferred using CLR and Kansas soil metatranscriptomic data. Each grey circle represents a gene (represented by an E.C. number) of the Kansas soil microbiome and each line represents an instance of high co-expression (a Z-score of greater than or equal to 4.20). (**B**) The same gene co-expression network but with genes associated with certain conditions highlighted. Red circles are genes associated with dry conditions, blue circles are those associated with wet conditions and yellow circles are genes associated with glycine-amendment conditions.

**Figure 3:**
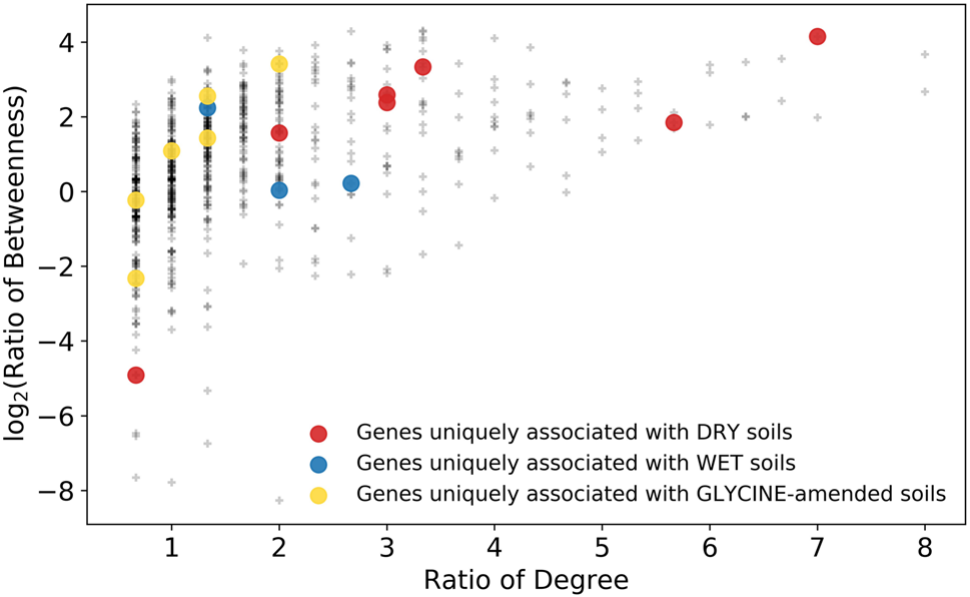
Centrality scores of genes associated with certain conditions of Kansas soil. Each gene in the network is shown as a colored dot on the graph. The centrality scores for each gene are shown on the y-axis and x-axis. The y-axis displays the log2 value of the ratio of Betweenness centrality for a given gene to the median Betweenness value for all genes. The x-axis displays the ratio of Degree centrality for a given gene to the median Degree value for all genes. Colored dots represent those genes associated with certain conditions. Red dots are genes associated with dry conditions, blue dots are those associated with wet conditions and yellow dots are genes associated with glycine-amendment conditions.

**Figure 4:**
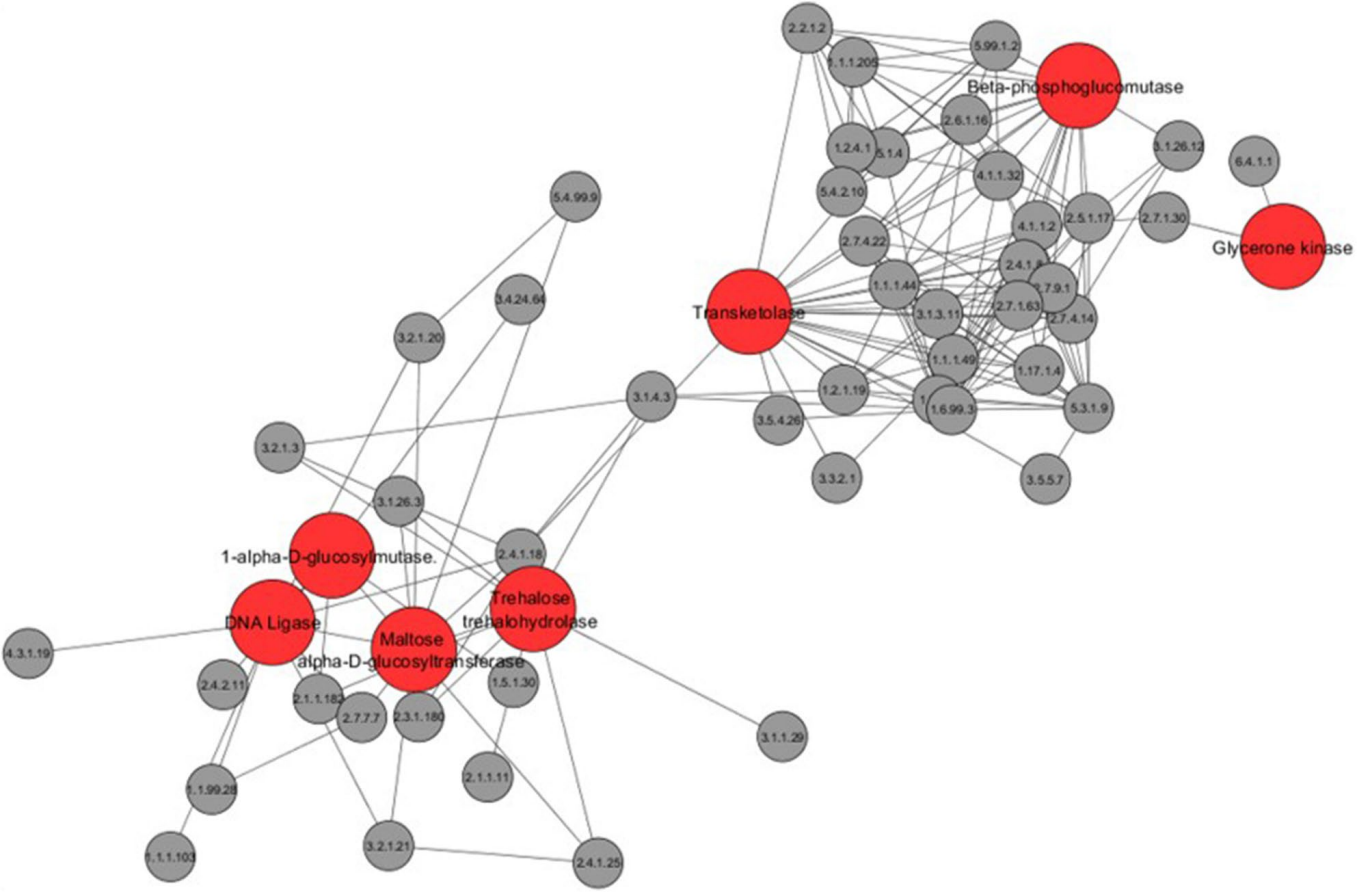
Network neighborhood of Dry associated genes. A subnetwork containing all genes that had an edge with at least one dry associated gene. Large red nodes are dry associated genes and functions for these are included as part of the figure. One gene, a prolyl aminopeptidase, was not connected with the cluster of other dry associated genes and is not included.

Compared to traditional statistical data analysis, metabolic network-based predictions above provided deeper insights into condition-specific biochemical reactions in soils. For example, our method predicted the synthesis of sugars such as trehalose and maltose in dry soils (and their degradation in wet soils)^9^, but metabolite (i.e., GC–MS) data showed no such changes across dry and wet conditions. With differential expression analysis, or more advanced feature selection methods, we could not fully predict the trehalose synthesis pathway as a biochemical signature for dry soils (Supp Table 2). By contrast, the integration of metabolites and genes using metabolic network models pin-pointed what specific pathways could be distinctively activated in soils across conditions.

**Table 2:** Centrality values of genes associated with glycine and moisture amended soil.

Associated condition	E.C Number	Function	Betweeness	Degree
Glycine	4.3.1.1	Aspartate ammonia-lyase	0.0320	6
Glycine	1.1.1.30	3-hydroxybutyrate dehydrogenase	0.0176	4
Glycine	3.1.1.75	Poly(3-hydroxybutyrate) depolymerase	0.0081	4
Glycine	2.6.1.66	Valine–pyruvate transaminase	0.0064	3
Glycine	1.1.1.36	Acetoacetyl-CoA reductase	0.0026	2
Glycine	3.1.1.22	Hydroxybutyrate-dimer hydrolase	0.0006	2
Glycine	2.8.3.5	3-oxoacid CoA-transferase	0.0000	2
Glycine	3.2.1.22	Alpha-galactosidase	0.0000	1
Wet	1.2.1.60	5-carboxymethyl-2-hydroxymuconic-semialdehyde dehydrogenase	0.0035	8
Wet	4.1.3.1	Isocitrate lyase	0.0142	4
Wet	1.1.1.42	Isocitrate dehydrogenase (NADP( +))	0.0031	6
Dry	5.4.99.15	(1- > 4)-alpha-D-glucan 1-alpha-D-glucosylmutase	0.0089	6
Dry	2.2.1.1	Transketolase	0.0532	21
Dry	5.4.99.16	Maltose alpha-D-glucosyltransferase	0.0303	10
Dry	6.5.1.1	DNA ligase (ATP)	0.0180	9
Dry	3.2.1.141	4-alpha-D-((1- > 4)-alpha-D-glucano)trehalose trehalohydrolase	0.0156	9
Dry	5.4.2.6	Beta-phosphoglucomutase	0.0108	17
Dry	3.4.11.5	Prolyl aminopeptidase	0.0157	6
Dry	2.7.1.29	Glycerone kinase	0.0001	2

### Inference of co-expression network of soil transcriptomic data

We next inferred a gene co-expression network for the soil microbiome by integrating data from all perturbation conditions. The network was inferred using CLR and the resulting gene networks were ranked (see Methods) before selecting a network of 1,096 nodes and 2,000 edges (Fig. 2A). Within this network each node represents a gene (annotated with an E.C. number) and each edge represents an instance of co-expression: included as edges in the network if they had a Z-score of at least ${Z}_{TH}$ (~ 4.20, i.e. ${Z}_{TH}$-folds standard deviations above the mean of all mutual information scores). As a final step the main connected cluster of the network was selected so that centrality analyses would be the most accurate. This resulted in a sub-network of 1,061 nodes and 1,978 edges.

Subsequently, we determined which genes occupied central positions in the network. The centrality of network genes can be measured by several metrics including how many edges a particular gene has (more edges equates to higher centrality) or how much a gene acts as a bridge between two separate clusters of genes (genes that occupy important bridging positions have higher centrality). Other studies have found that genes that have high centrality by either of these measures are critically important to the system^11,12^. We identified the most central genes in the networks inferred here (Fig. 2B). Two different measurements of centrality were applied: degree (number of edges) and betweenness (how much a gene acts as a bridge). Degree was used as a proxy for genes that are critically important to a small number of pathways the have many connections to other genes. Betweenness was used as a proxy for genes that may be involved in multiple different pathways and are linked to genes in disparate portions of the network. Genes of high centrality in the network are shown in Table 1 and include several genes involved in key metabolic pathways such as gluconeogenesis and starch and sucrose metabolism. Genes involved in respiration and with synthesis of, or resistance to, antibiotics were also highly central. One gene, encoding glycoaldehyde transferase, was of very high centrality when ranked by both betweenness (0.053, ranked 4th out of 1,061 genes) and degree (21, ranked 3rd out of 1,061 genes).

### Centralities of condition-specific genes and their functional relationships to other genes

As centrality can be used as a proxy for functional importance, we next aimed to determine if any of the genes that were associated with specific growth conditions occupied central positions in the network. All genes were graphed and their associated centrality values for both degree and betweenness were determined. This showed that genes associated with dry conditions occupied much higher centrality values compared to other genes, even those preferentially associated with either wet or glycine conditions (Fig. 3). The average betweenness value for genes in the network was 0.006 while ‘dry-associated genes' in the network had an average betweenness value of 0.017 (2.83-fold higher than average). The average degree value for genes in the network was 3.72 while ‘dry-associated genes' in the network had an average betweenness value of 9.375 (2.5-fold higher than average). Only two ‘dry-associated genes', EC 2.7.1.29 (glycerone kinase) and EC 3.4.11.5 (prolyl aminopeptidase) had betweenness and degree values that were lower than the average (Table 2). This finding contrasts with genes associated with wet or glycine-amended conditions. The three genes in the network that were associated with wet conditions had an average betweenness value of 0.007, only 1.1-fold higher than average, with 2/3 of the genes having below average betweenness, and an average degree value of 6 (1.6-fold higher than the average) (Table 2). Genes associated with glycine were of even lower centrality with eight genes in the network having an average betweenness value of 0.008, 1.36-fold higher than average, but with 4/8 genes showing lower than average betweenness. 'Glycine-associated genes' had an average degree value of 3 (lower than the average) with 5/8 of the genes having a below average degree value compared to all genes in the network (Table 2).

Networks present powerful ways to view not only which processes occupy central positions and are thus potentially 'important', but also how genes and processes are related to each other. Therefore, we next determined which genes were connected to the highly central genes associated with dry conditions. This was performed by forming a subnetwork consisting of genes that had an edge with at least one of the seven genes associated with dry conditions, excluding EC 3.4.11.5 which was not in proximity to other ‘dry-associated genes'. This subnetwork contained 55 genes (including the seven associated with dry conditions) with 178 edges between them (Fig. 4). Among these 55 genes, the following functions were enriched: biosynthesis of secondary metabolites (e.g. ansamycins, and siderophores [Isochorismatase, 3.3.2.1]) as well as carbon metabolism (e.g. starch and sucrose metabolism, pentose phosphate pathway and glycolysis/gluconeogenesis). These findings suggest that these processes may be important to the response of the soil community to dry conditions.

## Discussion

In recent years, multi-omics technologies have advanced to the point that they can now be used to help decipher functions carried out by complex soil microbial communities^27^. However, the resulting data are still computationally challenging to interpret due to the complexity and diversity of the data. Here, we demonstrated that successful integration of two modeling approaches to multi-omics data derived from soil that had been subjected to different environmental perturbations (wetting, desiccation or nutrient amendment) not only enabled prediction of unique genes and pathways that responded to each of the conditions, but also revealed their relationships with structural centralities. By combining two complementary modeling approaches (metabolic and gene network modeling) we were able to achieve a deeper understanding of the metaphenomic response of the soil microbial community to the specific perturbations.

Development of reliable computational network models poses a challenge due to intrinsic hurdles associated with collection of omics data from soil samples. In particular, metabolite extraction from soil can be affected by a number of variables not present in more controlled systems including soil pH, moisture, temperature, and particle size. Chemical functional groups of metabolites can sorb to hydrophobic/philic particles in soil and temperature and pH can influence solubility and extraction. All of this means that metabolites with different chemical moieties might not be extracted and analyzed equally. Due to these challenges, we conservatively used only a subset of metabolites that were identified in different conditions. While rigorous evaluation of the level of bias was not possible, we confirmed that (1) these metabolites were compounds commonly detected in environmental samples, and (2) they were almost identical across perturbation conditions. This implies that prediction of “condition-specific” genes/reactions was primarily affected by differential gene expression profiles rather than metabolite data. However, successful prediction of those signature molecules required inclusion of metabolite data due to their role as hard constraints on metabolic network models. Integration of both transcriptomic and metabolomic data therefore complemented each other, consequently leading us to minimize challenges in obtaining unbiased data collection.

The analysis of gene expression networks provided new insight that could not be obtained by metabolic network modeling alone. Previous studies of gene co-expression network structure have revealed that centrality can be a proxy for functional importance^10,11^, and that there is a significant overlap between genes in bacterial co-expression networks that occupy highly central positions and those that are part of central metabolic pathways that are crucial for growth^12^. Here, we find that (1) the unique genes associated with certain conditions occupy various centralities in our gene co-expression network and (2) dry-associated genes occupy more central positions in the network than other condition-specific genes.

The observation that dry-associated genes are more central in our network may suggests such pathways are critical to soil microbiomes as they respond to a number of other conditions as well. It is important to note that our gene co-expression network is made from data representing several different conditions, therefore centrality values are derived from a model that shows the overall collective response to all of these conditions. Drought conditions not only lead to a great deal of environmental stress on the soil microbiome, but also increase other kinds of stress such as the lack of nutrients (as they are no longer soluble), increase in salt stress, etc. Other studies have also shown that lack of water leads to larger changes in the soil microbiome compared to other stresses^28^, perhaps explaining the central position that drought response occupies. These results indicate that the ability to respond to drought stress is central and important, more so than the response to excessive water or influxes of carbon.

We also showed evidence that drought processes are critically important based on their links within the network to other pathways. Processes that are linked in networks reflect points of coordination and similar expression between these processes. The fact that dry-associated genes are linked to genes involved in central metabolic pathways (pentose phosphate, glycolysis/gluconeogenesis) strongly indicates that processes responding to dry conditions are central to the functioning of the soil microbiome. Dry-associated genes were also linked to siderophore genes suggesting that these processes (drought response, siderophore production) are correlated. Siderophore production has been linked to the responses of plants and bacteria during drought stress^29–31^ and while no plants were included in these studies soil samples were from fields where plants were present, suggesting that bacterial processes linked to plant–microbe interactions are correlated with drought responses.

The studies here lead to two general conclusions: (1) a combined approach of multiple modeling strategies provides a new understanding of soil biochemistry (such as the relationships between gene’s structural centrality and condition specificity) that cannot be obtained by each approach in isolation, and (2) dry-associated genes occupy central and important positions in a network model of the soil microbiome, suggesting that for this soil, it was critical for the soil microorganisms to be able to respond to soil drying, as would be expected under drought. Future studies will make use of additional -omics data (such as proteomics) to increase the value of networks of models of microbiomes. The use of modeling approaches, specifically a combinatorial approach shown here, is a powerful way to interpret large amounts of data describing complex systems. The hypotheses generated can be tested experimentally in natural soil systems, providing new information about how these systems respond to a changing environment, such as expected to occur with climate change.

## Methods

### Soil samples and perturbation experiments

Soil samples were collected from the Konza Prairie Biological Station (KPBS), as previously described^9, 32^. In brief, composite samples (0–15 cm) were obtained from three field locations (sites A, B and C) representing a natural hydrologic gradient. The soil was frozen in liquid nitrogen in the field and shipped frozen on dry ice to the Pacific Northwest National Laboratory (PNNL). Immediately upon receipt at PNNL, the soil was quickly thawed and the individual field replicates were immediately sieved (< 2 mm) and proportioned into ~ 50 g aliquots in eighteen 50 ml Falcon Tubes per field location (resulting in 18 identical reps per site A, B and C). The soil aliquots were stored frozen (6 months to 1.5 years) at − 80 C until used in perturbation experiments. Three replicates of each field location were subjected to two different types of perturbations: nutrient (glycine) addition or soil moisture stress (wetting to saturation or drying). Glycine was chosen as a nutrient amendment because it is a common root exudate that the soil microbiome is likely to be exposed to in soils^21,22^. Soil samples were thawed and pre-incubated at 21 °C overnight before the onset of the respective perturbation experiments. For nutrient addition, a glycine solution (10 mM) was added to 10 g field-moist soil in 50 mL falcon tubes to a final concentration of 0.027 mmol g^−1^ dry weight soil and mixed using sterile pipette tips. Nine microcosms (3 sites × 3 replicates) were supplemented with glycine and are referred to as "Gly-positive" samples and another 9 were maintained as controls after adjusting with de-ionized water. The 18 microcosms thus constructed were incubated at 21 °C in the dark for 48 h, the period during which the highest respiration activity was measured^9^. In a separate experiment using the same soil samples, herein referred to as the soil moisture perturbation, soils were similarly pre-incubated and subjected to three moisture conditions: saturated, air-dried to constant weight or maintained at field-moist or control conditions in triplicate microcosms, as previously described^9^. At the end of the respective perturbation experiments, subsamples from each replicate microcosm were collected and analyzed to determine which soil microbial community genes were expressed (metatranscriptomes) and the metabolic compositions of the soil communities. Details of ribonucleic acid (RNA) and metabolite extractions (using MPLEX), sequencing the metatranscriptome and gas chromatography-mass spectrometry (GC–MS) analysis of the metabolome, and raw data processing were previously described^9^. We note that metatranscriptomes from soil B that had undergone moisture perturbations could not be obtained due to challenges with obtaining sufficient RNA^9^.

### Prediction of active metabolic reactions in each condition using metabolic network models

The MEMPIS algorithm^9^ was applied to the multi-omics datasets (i.e., genes and metabolites) to identify condition-specific pathways or subnetworks of reactions. To reiterate, both metabolite and gene expression data were available for the control and treatment samples, which included dry soils A and C, wet soils A and C, and glycine-amended soils A, B, and C. A complete biochemical reaction map obtained from the comprehensively curated KEGG database was used as a master metabolic network to incorporate metabolites and genes. While the master metabolic network was generic, the pathways resulting from network-omics integration were condition-specific through the combination of site-specific omics profiles. The MEMPIS algorithm identified *minimal* subnetworks that connect 1) all identified metabolites and 2) over-expressed genes that satisfy two prescribed thresholds for fold changes and adjusted *p*-values in each perturbation against its control sample.

### Data-driven feature selection

For comparison to the metabolic network-based identification of condition-specific genes/reactions, data-driven feature selection methods were performed to extract key signatures from the metatranscriptomic data that effectively represented each experimental condition. The recursive feature elimination and cross-validated selection was performed using the tree-based estimators to differentiate dry, wet, glycine and control conditions, and implemented based on a python package, scikit-learn (https://scikit-learn.org/). We performed PCA and ANOVA tests using the same python package to extract statistically significant features. Features identified by these selection methods were considered statistically significant if the adjusted *p* values < 0.05 (in the ANOVA test).

### Gene co-expression networks

Gene expression data collected from the two perturbation experiments were used with the Context Likelihood of Relatedness (CLR)^33^ program to infer a network where genes were nodes and edges were instances of high co-expression between nodes. CLR was run using default settings with the output being a matrix of Z-scores of mutual information values between all gene pairs. Gene pairs with higher Z-scores are considered to be more tightly co-expressed. The weighted Z-score matrix was converted to an unweighted matrix that replaced all Z-scores with either a zero (if it was below our cutoff for an edge) or a one (if it was above our cutoff). A critical decision point in inferring an unweighted matrix for network analysis is the choice of cutoff used to define an edge in the network. Here, we tested several cutoffs and chose 4.20, meaning that genes with a mutual information score that was at least 4.20 standard deviations above the mean of all mutual information scores in the matrix were connected by an edge in the network. This cutoff was chosen because it was high enough to ensure that only biologically relevant edges were included in our results (a score of 4.2 corresponds to a *p* value of < 5E-5) and because it led to a network with significant structure for analysis. The resulting network has a node degree distribution that fit a power law (R^2^ value of 0.935), a common feature of scale-free biological networks^34^. Resulting unweighted networks were viewed in Cytoscape^35^. Centrality values, betweenness and degree, were also calculated using Cytoscape. Annotations for genes were pulled from KEGG^36^.

## Supplementary information

Supplementary file1 (DOCX 501 kb)

## Data Availability

The source code of this work will be made available upon request to the corresponding author.
