# Supplementary material for: Integrated network modeling approach defines key metabolic responses of soil microbiomes to perturbations
Source: Sci Rep. 2020 Jul 2;10:10882. doi: 10.1038/s41598-020-67878-7 (PMC7331712; doi:10.1038/s41598-020-67878-7)
Supplement: Supplementary file 1 — Supplementary file1 (DOCX 501 kb) [file 41598_2020_67878_MOESM1_ESM.docx]

**Supplementary Information**

**Integrated network modeling approach defines key metabolic responses of soil microbiomes to perturbations**

Ryan S. McClure^1†^, Joon-Yong Lee^1†^, Taniya Roy Chowdhury^1‡^, Eric M. Bottos^1‡^, Richard Allen White III^1‡^, Young-Mo Kim^1^, Carrie D. Nicora^1^, Thomas O. Metz^1^, Kirsten S. Hofmockel^1,2^, Janet K. Jansson^1^, and Hyun-Seob Song^1,3,4*^

^1^Biological Sciences Division, Pacific Northwest National Laboratory, Richland WA, USA; ^2^Department of Ecology, Evolution and Organismal Biology, Iowa State University, Ames, Iowa, USA; ^3^Department of Biological Systems Engineering, University of Nebraska-Lincoln, Lincoln, Nebraska, USA; ^4^Nebraska Food for Health Center, Department of Food Science and Technology, University of Nebraska-Lincoln, Lincoln, Nebraska, USA

^‡^Current address: Taniya RoyChowdhury, Environmental Science and Technology, Department, University of Maryland, College Park, Maryland, USA; Eric M. Bottos, Department of Biological Sciences, Thompson Rivers University, Kamloops BC, Canada; Richard Allen White III, Department of Plant Pathology, Washington State University, Pullman, Washington, USA

*Corresponding Author: [hsong5@unl.edu](mailto:hsong5@unl.edu)

**Supplementary Figures 1, 2, 3, and 4**

**Supplementary Tables 1 and 2**


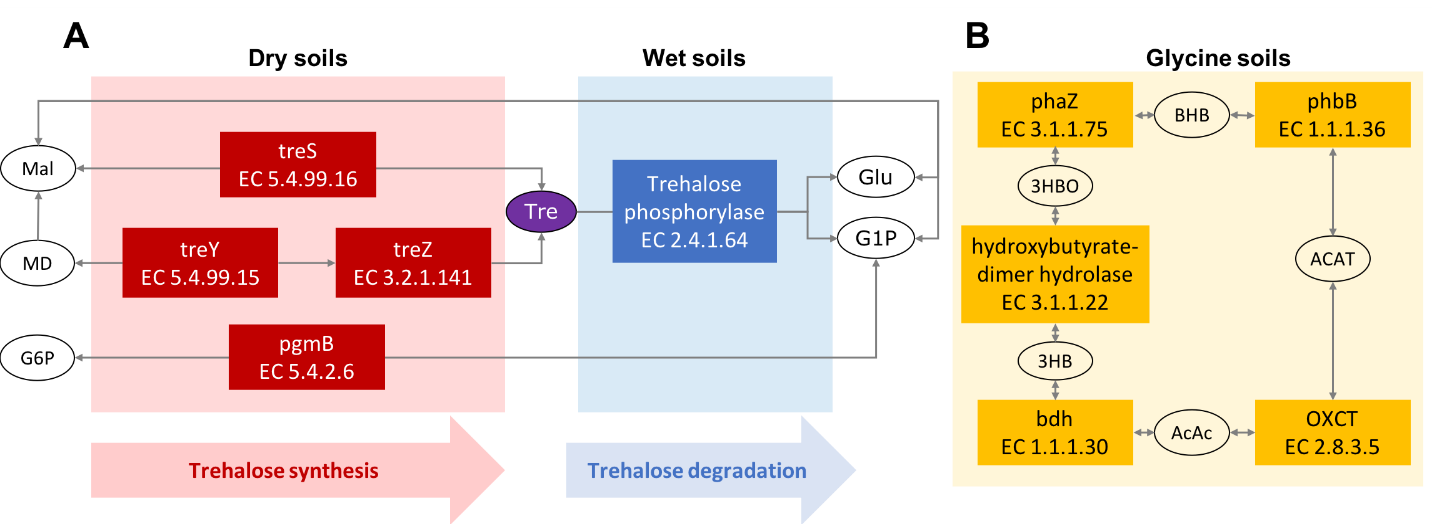


**Supplementary Figure 1. Predicted active reactions in different conditions. (A)** Dry and wet signature genes in the starch and sucrose metabolism pathway (Tre: Trehalose, Glu: D-Glucose, Mal: Maltose, MD: Maltodextrin, G6P: D-Glucose-6P, and G1P: β-D-Glucose-1P) and **(B)** Glycine signature genes in the butanoate metabolism pathway (BHB: Beta-hydroxybutyrate, AcAc: Acetoacetate, ACAT: Acetoacetyl-CoA, 3HBO: (R)-3-((R)-3-Hydroxybutanoyloxy)butanoate, 3HB: (R)-3-Hydroxybutanoate.

**
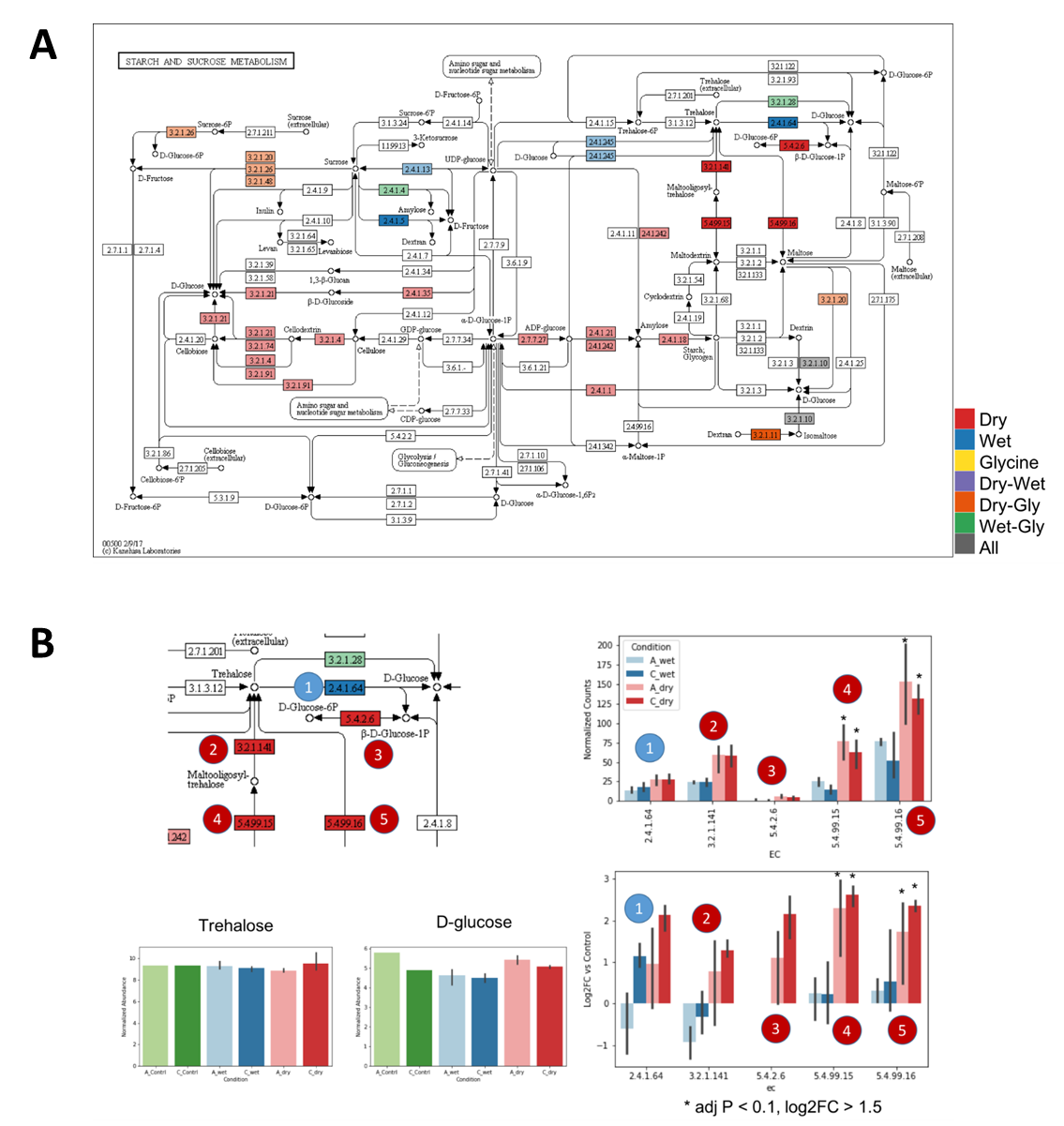
**

**Supplementary Figure 2. Starch and sucrose metabolism pathway:** (A) Condition-specific signature genes in the starch and sucrose metabolism pathway. Red, blue, and yellow colors indicate the dry, wet, and glycine signature genes uniquely predicted in individual condition, respectively. And the mixtures of two colors (i.e., purple, orange, and green) describe that these genes are commonly predicted in two different conditions. Black colored boxes represent the common genes predicted in all three conditions. Thicker colors indicate that these genes are consistently predicted in the different soils of the same condition. (B) Metatranscriptome and metabolome data of dry and wet signature genes. The left-top pane illustrates the zoomed-in sub-pathway of the trehalose synthesis and degradation and numbers on the predicted signature genes. The left-bottom pane illustrates the abundance levels of the trehalose and D-glucose across soil samples. The Right panes describe the normalized counts (top) and the log-scaled fold changes (bottom) against the control samples for signature genes.

**
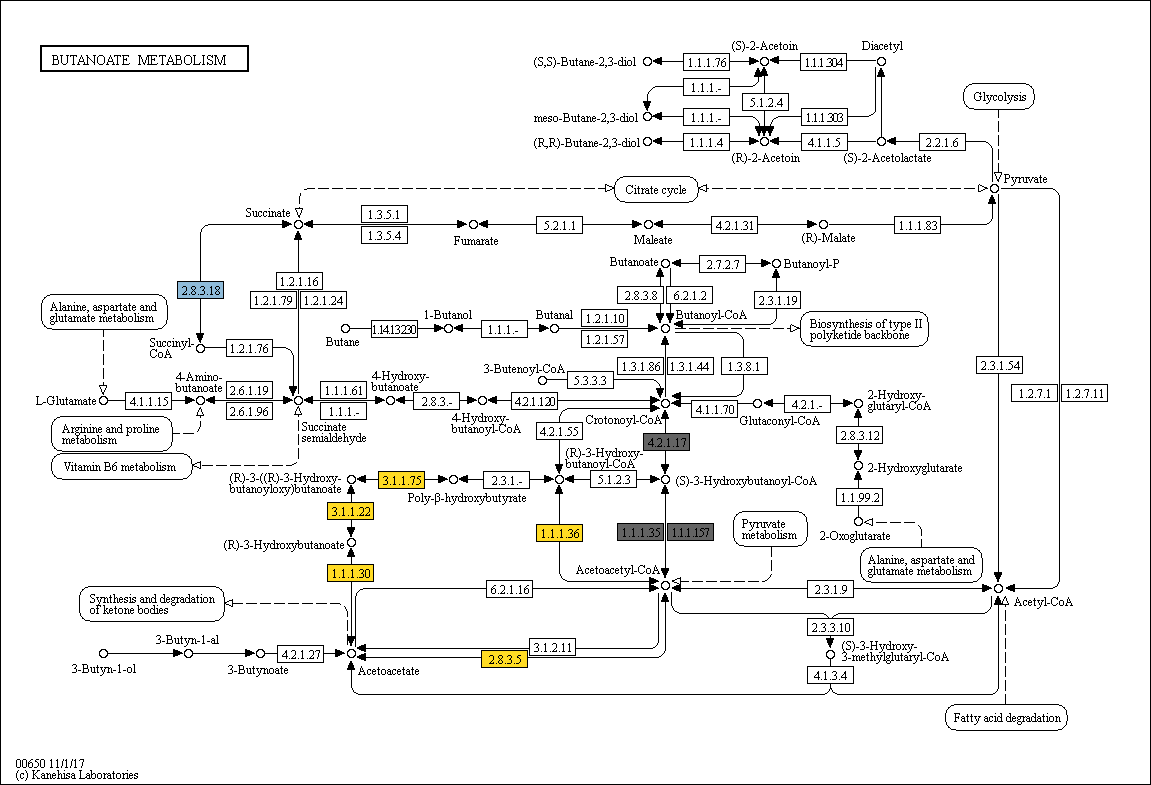
**

**Supplementary Figure 3. Butanoate metabolism pathway.** There are 5 glycine signature genes (yellow), 3 genes (black) predicted in all conditions, and 1 genes (blue) predicted in the soil C and wet condition.

**
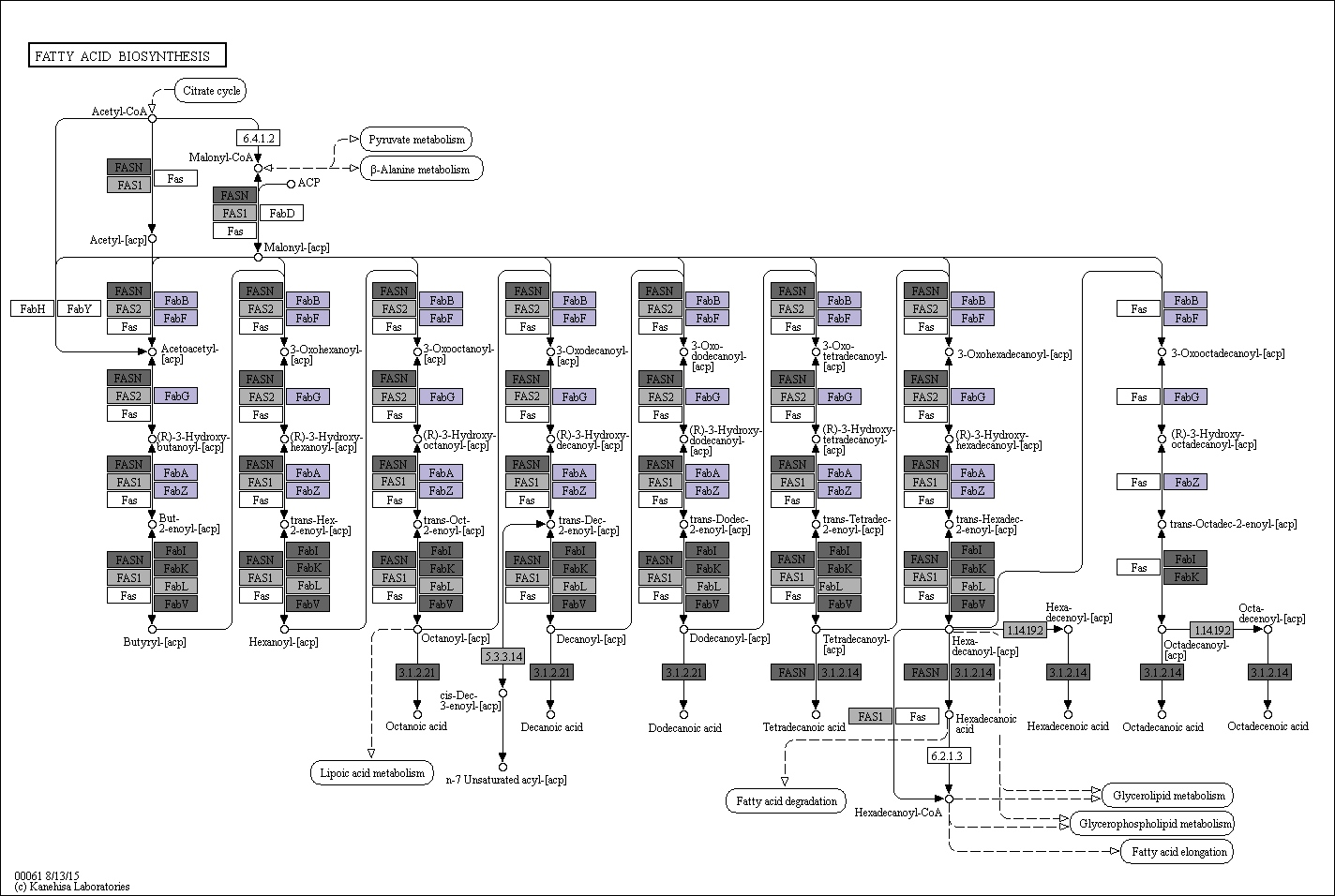
**

**Supplementary Figure 4. Fatty acid biosynthesis pathway.** The enoyl-[acyl-carrier protein] reductase I (fabI, EC 1.3.1.9, 1.3.1.10) , enoyl-[acyl-carrier protein] reductase II (fabK, EC 1.3.1.9) , enoyl-[acyl-carrier protein] reductase / trans-2-enoyl-CoA reductase (NAD+) (fabV, EC:1.3.1.9 1.3.1.44), medium-chain acyl-[acyl-carrier-protein] hydrolase (MCH, EC 3.1.2.21), fatty acyl-ACP thioesterase B (FATB, EC:3.1.2.14 3.1.2.21), fatty acid synthase (FASN, EC 2.3.1.85) are commonly predicted in all 7 soil samples.

**Supplementary Table 1. List of condition-specific genes predicted from MEMPIS.**

**Supplementary Table 2. Selected features from the recursive feature elimination and cross-validated selection in moisture conditions.** These 31 features were selected from the recursive feature elimination and cross-validated selection. The green colored background means the 16 features with adj p < 0.05 (ANOVA test). The data-driven feature selection presents 1 dry gene (red text) and 1 wet gene (blue text).

| EC | AwetRep1 | AwetRep2 | AwetRep3 | CwetRep1 | CwetRep2 | CwetRep3 | AdryRep1 | AdryRep2 | AdryRep3 | CdryRep1 | CdryRep2 | CdryRep3 | pca_len | pval | adjPval | fscores | Ranking |
| --- | --- | --- | --- | --- | --- | --- | --- | --- | --- | --- | --- | --- | --- | --- | --- | --- | --- |
| 1.3.3.5 | 27.50 | 40.00 | 40.95 | 25.45 | 41.25 | 45.00 | 11.67 | 9.41 | 15.71 | 14.62 | 11.43 | 6.00 | 0.08 | 0.00 | 0.01 | 49.36 | 1 |
| 1.14.14.7 | 3.75 | 3.08 | 2.86 | 2.73 | 1.25 | 2.50 | 0.00 | 0.00 | 1.43 | 0.00 | 0.00 | 0.00 | 0.08 | 0.00 | 0.01 | 39.54 | 1 |
| 1.2.2.2 | 13.75 | 4.62 | 6.67 | 8.18 | 2.50 | 2.50 | 69.17 | 48.82 | 30.00 | 26.15 | 60.95 | 36.00 | 0.13 | 0.00 | 0.01 | 46.73 | 1 |
| 3.4.11.15 | 7.50 | 4.62 | 6.67 | 5.45 | 2.50 | 5.00 | 19.17 | 28.82 | 18.57 | 11.54 | 13.33 | 22.00 | 0.07 | 0.00 | 0.01 | 40.46 | 1 |
| **5.4.99.15** | **30.00** | **20.00** | **24.76** | **20.00** | **10.00** | **15.00** | **97.50** | **78.24** | **54.29** | **43.08** | **67.62** | **78.00** | **0.09** | **0.00** | **0.01** | **42.37** | **1** |
| 4.1.2.43 | 22.50 | 15.38 | 15.24 | 19.09 | 15.00 | 17.50 | 9.17 | 2.35 | 8.57 | 5.38 | 4.76 | 6.00 | 0.06 | 0.00 | 0.01 | 33.54 | 1 |
| 1.2.1.65 | 1.25 | 0.00 | 0.00 | 0.00 | 0.00 | 0.00 | 3.33 | 2.94 | 2.86 | 2.31 | 5.71 | 1.00 | 0.08 | 0.00 | 0.01 | 32.41 | 1 |
| 1.1.1.274 | 8.75 | 4.62 | 6.67 | 10.00 | 3.75 | 1.25 | 22.50 | 17.65 | 15.71 | 20.77 | 29.52 | 29.00 | 0.09 | 0.00 | 0.02 | 26.83 | 1 |
| 3.5.99.4 | 0.00 | 0.00 | 1.90 | 1.82 | 0.00 | 0.00 | 10.83 | 2.94 | 4.29 | 4.62 | 6.67 | 3.00 | 0.09 | 0.00 | 0.02 | 25.89 | 1 |
| **1.2.1.60** | **185.00** | **321.54** | **555.24** | **230.91** | **412.50** | **307.50** | **47.50** | **54.12** | **248.57** | **42.31** | **47.62** | **58.00** | **0.11** | **0.00** | **0.02** | **24.88** | **1** |
| 3.1.4.4 | 8.75 | 1.54 | 7.62 | 1.82 | 6.25 | 2.50 | 28.33 | 30.00 | 14.29 | 11.54 | 20.00 | 20.00 | 0.10 | 0.00 | 0.02 | 24.98 | 1 |
| 1.1.99.13 | 3.75 | 10.77 | 5.71 | 7.27 | 5.00 | 7.50 | 3.33 | 0.00 | 1.43 | 0.77 | 1.90 | 0.00 | 0.09 | 0.00 | 0.02 | 23.67 | 1 |
| 2.4.1.230 | 7.50 | 1.54 | 1.90 | 2.73 | 2.50 | 8.75 | 22.50 | 24.12 | 21.43 | 8.46 | 10.48 | 16.00 | 0.10 | 0.00 | 0.03 | 21.90 | 1 |
| 3.4.24.28 | 8.75 | 9.23 | 11.43 | 5.45 | 23.75 | 7.50 | 23.33 | 27.06 | 20.00 | 40.77 | 64.76 | 41.00 | 0.07 | 0.00 | 0.03 | 20.34 | 1 |
| 1.7.3.3 | 15.00 | 21.54 | 23.81 | 26.36 | 41.25 | 17.50 | 10.83 | 4.71 | 4.29 | 11.54 | 11.43 | 11.00 | 0.05 | 0.00 | 0.04 | 18.68 | 1 |
| 2.7.1.56 | 3.75 | 1.54 | 8.57 | 2.73 | 1.25 | 2.50 | 7.50 | 6.47 | 12.86 | 7.69 | 10.48 | 13.00 | 0.06 | 0.00 | 0.04 | 16.95 | 1 |
| 1.13.12.4 | 0.00 | 0.00 | 0.00 | 0.00 | 0.00 | 0.00 | 3.33 | 0.00 | 2.86 | 1.54 | 0.95 | 1.00 | 0.04 | 0.00 | 0.05 | 15.36 | 1 |
| 3.1.1.43 | 5.00 | 3.08 | 5.71 | 3.64 | 1.25 | 3.75 | 1.67 | 0.59 | 0.00 | 0.77 | 0.00 | 3.00 | 0.05 | 0.01 | 0.08 | 11.72 | 1 |
| 4.2.1.113 | 7.50 | 1.54 | 4.76 | 4.55 | 2.50 | 2.50 | 1.67 | 2.35 | 0.00 | 0.77 | 0.00 | 1.00 | 0.04 | 0.01 | 0.08 | 11.79 | 1 |
| 2.6.1.3 | 8.75 | 6.15 | 5.71 | 13.64 | 5.00 | 6.25 | 4.17 | 4.71 | 2.86 | 0.77 | 2.86 | 4.00 | 0.04 | 0.01 | 0.09 | 10.86 | 1 |
| 6.4.1.1 | 138.75 | 261.54 | 576.19 | 85.45 | 152.50 | 43.75 | 39.17 | 39.41 | 85.71 | 39.23 | 33.33 | 48.00 | 0.10 | 0.01 | 0.12 | 9.68 | 1 |
| 3.1.2.12 | 2.50 | 1.54 | 7.62 | 3.64 | 1.25 | 0.00 | 0.00 | 0.59 | 0.00 | 0.77 | 0.00 | 0.00 | 0.07 | 0.01 | 0.14 | 8.85 | 1 |
| 3.4.24.29 | 3.75 | 3.08 | 1.90 | 3.64 | 3.75 | 5.00 | 2.50 | 0.59 | 0.00 | 0.00 | 1.90 | 3.00 | 0.04 | 0.02 | 0.14 | 8.39 | 1 |
| 4.4.1.22 | 8.75 | 0.00 | 4.76 | 0.91 | 8.75 | 6.25 | 0.00 | 0.59 | 0.00 | 1.54 | 1.90 | 0.00 | 0.11 | 0.03 | 0.20 | 6.14 | 1 |
| 1.7.1.13 | 6.25 | 7.69 | 9.52 | 9.09 | 1.25 | 7.50 | 4.17 | 0.00 | 2.86 | 0.77 | 3.81 | 3.00 | 0.07 | 0.03 | 0.21 | 5.99 | 1 |
| 3.4.24.25 | 38.75 | 13.85 | 2.86 | 3.64 | 2.50 | 6.25 | 0.00 | 1.76 | 4.29 | 1.54 | 2.86 | 2.00 | 0.06 | 0.04 | 0.23 | 5.70 | 1 |
| 2.7.7.38 | 2.50 | 13.85 | 7.62 | 11.82 | 3.75 | 10.00 | 5.00 | 4.71 | 1.43 | 1.54 | 5.71 | 3.00 | 0.05 | 0.05 | 0.27 | 4.93 | 1 |
| 3.5.1.26 | 5.00 | 1.54 | 0.95 | 1.82 | 1.25 | 0.00 | 0.00 | 1.18 | 0.00 | 0.00 | 0.00 | 1.00 | 0.03 | 0.05 | 0.27 | 4.92 | 1 |
| 3.1.8.1 | 7.50 | 0.00 | 0.95 | 5.45 | 3.75 | 3.75 | 0.83 | 1.76 | 0.00 | 1.54 | 1.90 | 1.00 | 0.07 | 0.15 | 0.42 | 2.50 | 1 |
| 4.3.1.4 | 2.50 | 0.00 | 0.95 | 1.82 | 3.75 | 1.25 | 3.33 | 0.59 | 0.00 | 0.00 | 0.95 | 0.00 | 0.06 | 0.19 | 0.47 | 1.98 | 1 |
| 2.7.1.45 | 2.50 | 1.54 | 3.81 | 3.64 | 3.75 | 2.50 | 4.17 | 3.53 | 0.00 | 1.54 | 2.86 | 0.00 | 0.02 | 0.19 | 0.48 | 1.93 | 1 |
